# An Outperforming Artificial Intelligence Model to Identify Referable Blepharoptosis for General Practitioners

**DOI:** 10.3390/jpm12020283

**Published:** 2022-02-15

**Authors:** Ju-Yi Hung, Ke-Wei Chen, Chandrashan Perera, Hsu-Kuang Chiu, Cherng-Ru Hsu, David Myung, An-Chun Luo, Chiou-Shann Fuh, Shu-Lang Liao, Andrea Lora Kossler

**Affiliations:** 1 Ophthalmology, Byers Eye Institute, Stanford University School of Medicine, 2452 Watson Court, Palo Alto, CA 94303, USA; hungjuyi@gmail.com (J.-Y.H.); gosienna@gmail.com (K.-W.C.); chandrashan@gmail.com (C.P.); david.myung@stanford.edu (D.M.); 2 Department of Computer Science and Information Engineering, National Taiwan University, Taipei 10617, Taiwan; fuh@csie.ntu.edu.tw; 3 Department of Biomedical Engineering, National Cheng Kung University, Tainan City 70101, Taiwan; 4 Computer Science, Stanford University, Stanford, CA 94305, USA; hsu-kuang.chiu@alumni.stanford.edu; 5 Ophthalmology, Tri-Service General Hospital, National Defense Medical Center, Taipei 114, Taiwan; josephinesheu@gmail.com; 6 Department of Electronic and Optoelectronic System Research Laboratories, Industrial Technology Research Institute, Hsinchu 31040, Taiwan; anchunluo@itri.org.tw; 7 Ophthalmology, National Taiwan University Hospital, Taipei 100, Taiwan; 8 College of Medicine, National Taiwan University, Taipei 10617, Taiwan

**Keywords:** artificial intelligence, blepharoptosis, general practitioners, computer-aided diagnosis (CAD)

## Abstract

The aim of this study is to develop an AI model that accurately identifies referable blepharoptosis automatically and to compare the AI model’s performance to a group of non-ophthalmic physicians. In total, 1000 retrospective single-eye images from tertiary oculoplastic clinics were labeled by three oculoplastic surgeons as having either ptosis, including true and pseudoptosis, or a healthy eyelid. A convolutional neural network (CNN) was trained for binary classification. The same dataset was used in testing three non-ophthalmic physicians. The CNN model achieved a sensitivity of 92% and a specificity of 88%, compared with the non-ophthalmic physician group, which achieved a mean sensitivity of 72% and a mean specificity of 82.67%. The AI model showed better performance than the non-ophthalmic physician group in identifying referable blepharoptosis, including true and pseudoptosis, correctly. Therefore, artificial intelligence-aided tools have the potential to assist in the diagnosis and referral of blepharoptosis for general practitioners.

## 1. Introduction

Blepharoptosis, also known as ptosis, is the drooping or inferior displacement of the upper eyelid. Ptosis can obstruct the visual axis and affect vision and can be a presenting sign of a serious medical disorder, such as ocular myasthenia [1], third cranial nerve palsy [2], or Horner syndrome [3]. It is important for general practitioners to accurately diagnosis ptosis to assist in decision making for referral and work up when necessary. Ptosis is diagnosed by using a ruler and light source to measure the distance between the pupillary light reflex and the upper eyelid margin (margin reflex distance 1, or MRD1) with the eyes in the primary position [4]. With low repeatability and reproducibility in measuring eyelid landmarks and the effect of learning curves [5,6], accurately recognizing ptosis is challenging especially for non-ophthalmologists. Therefore, an automated tool for ptosis diagnosis may be useful for general practitioners.

Currently, artificial intelligence (AI)-aided diagnostic tools play a promising role in the automatic detection of certain diseases, such as diabetic retinopathy [7] and skin cancer [8] from retinal fundus and skin images, respectively. Convolutional neural network (CNN)-based deep learning methods, a subset of machine learning techniques, have been the state of the art in AI for years, leading to enhanced performance in various medical applications [9]. It requires less supervision and uses an end-to-end learning mechanism to map raw inputs, such as image pixels, to outputs without human-directed manipulation of data [10]. The image-to-classification approach in one classifier replaces the multiple steps of previous image analysis methods [11].

In a previous study [12], a variety of CNN architectures, such as VGG-16 [13], ResNet [14], and DenseNet [15], diagnosed true blepharoptosis without any inputs of eyelid measurements from a clinical photograph, achieving a high accuracy of 83.3% to 88.6%. In this study, we further trained an AI model using the VGG-16 architecture with larger and more diverse datasets to accurately diagnose blepharoptosis and compared the AI model’s performance to a group of non-ophthalmic physicians. Our goal was to determine if our AI model could outperform physicians to support the need for an AI tool to diagnose blepharoptosis.

## 2. Materials and Methods

### 2.1. Image Preparation

Original photographs, taken by a hand-held digital camera (Canon DIGITAL IXUS 950 IS) at a tertiary oculoplastic clinic of adult patients over 20 years old, were retrospectively collected over the past 20 years for surgical evaluation. A total of 1000 images were used in this study. IRB approval was granted for this study by Stanford University, and the research was conducted in accordance with National Taiwan University IRB protocol. 

In order to crop a standardized image of a single eye, OpenFace [16], an open-source package, was utilized to identify major facial landmarks in each photograph. Cropped single-eye images were 400 × 600 pixels individually and were then resized to 200 × 300 pixels, matching the input size, which was ready to be used in the CNN architectures.

### 2.2. Inclusion and Exclusion

**Figure 1:**
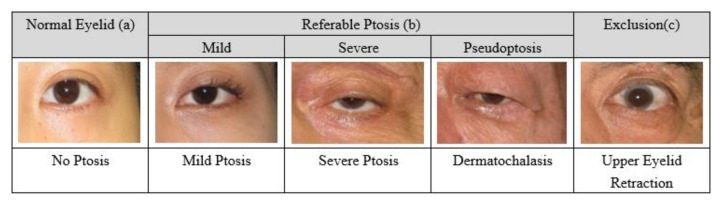
Healthy (**a**), referable ptosis (**b**), and excluded (**c**) group (right eyes).

After cropping, the photographs involved only the periocular region of a single eyes Figure 1. The appearance of a healthy eyelid is illustrated in Figure 1a. The referable ptosis group included mild ptosis, severe ptosis, and pseudoptosis (dermatochalasis), a condition in which excess upper eyelid skin overhangs the eyelid margin Figure 1b. Upper eyelid retraction was excluded Figure 1c. Poor quality images, including uncentered visual fixation, uneven curves of the upper eyelids, and blurred upper eyelid margins due to dense eyelashes, were excluded. A total of 1000 images were evaluated and 218 images were removed, leaving 782 images for use in this study.

The brow region was not included in the photographs; therefore, brow ptosis was not excluded. Exact measurements, such as margin to reflex distance 1 (MRD-1), MRD-2 [17,18], levator function [19], or palpebral aperture [20], were not provided. The condition of the other eye and the history of the patients were withheld.

### 2.3. Annotations for the Ground Truth

**Figure 2:**
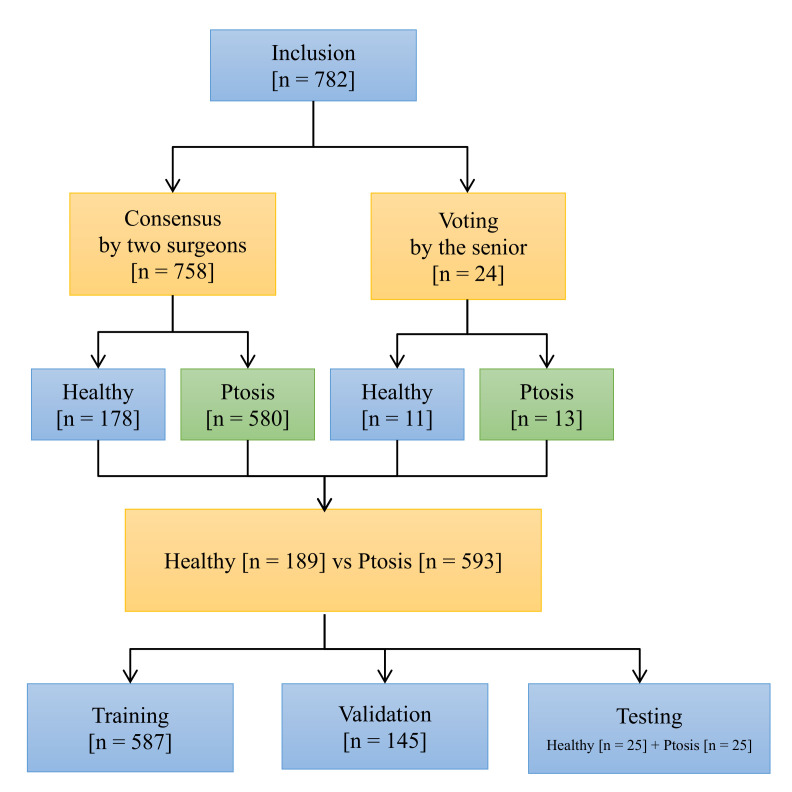
Flowchart for data labeling.

Two labelers, both oculoplastic surgeons, achieved an 82% consensus rate in discussion meetings. The major reasons for their disagreements were decisions about healthy eyelids and mild ptosis. To lessen spectrum bias, a third senior oculoplastic surgeon, as an arbiter, yielded the decisive answer for these disagreements, which included 24 images. Figure 2 shows the voting system, with 593 images (accounting for 75%) in the referable ptosis group and 189 (25%) images in the healthy group.

### 2.4. Data Allocation for Training, Validation, and Testing

A total of 50 images, including 25 healthy eyelids and 25 ptotic eyelids, were randomly selected into testing datasets. The same testing datasets were used to test the AI model and the physician group. The rest of the photographs were then divided into training and validation datasets with the ratio of 8:2 Table 1.

### 2.5. Model Architecture and Training

VGG-16 was used as the base structure [13,21]. The last few layers of VGG-16′s architecture were replaced with a global max pooling layer followed by fully connected layers and a sigmoid function for our binary classification problem. In order to reduce memory usage, the size of the input images was adjusted to 200 × 300 pixels. The details of our model architecture can be seen in Table 2.

### 2.6. Transfer Learning and Data Augmentation

Transfer learning was performed by importing weights trained on ImageNet [22]. Tensorflow 2.0 with Keras was used as our training framework. For learning rate optimization, Adam optimizer was applied [21]. Data augmentation was also used to prevent overfitting. The transformations of photographs included:

- Images flipped horizontally;
- Random image rotations of up to 15 degrees;
- Random zooms in or out between the range of 90% to 120%;
- Adjusted brightness/contrast by 50%;
- Images shifted horizontally or vertically by 10%.

### 2.7. Testing in Non-Ophthalmic Physician Group

Three specialists, one each from emergency medicine, neurology, and family medicine, were tested on behalf of the non-ophthalmic physician group. The clinical experience of each of the three physicians was over five years. The same testing set, including 25 healthy eyelids and 25 ptotic eyelids, was given to the group to distinguish ptotic eyelids from healthy eyelids. No other information, such as MRD-1 measurements, the condition of the other eye, or patient histories, were provided. Moreover, no further training on blepharoptosis diagnosis was given. The decision making relied on each physician’s personal background knowledge. 

## 3. Results

There were 45 correct predictions, including 22 healthy and 23 ptosis answers, by the CNN model from a total of 50 testing images. The accuracy of the AI model was 90%, with a sensitivity of 92% and a specificity of 88%. Three false positives and two false negatives were found.

### 3.1. Confusion Matrix and ROC Curve

**Figure 3:**
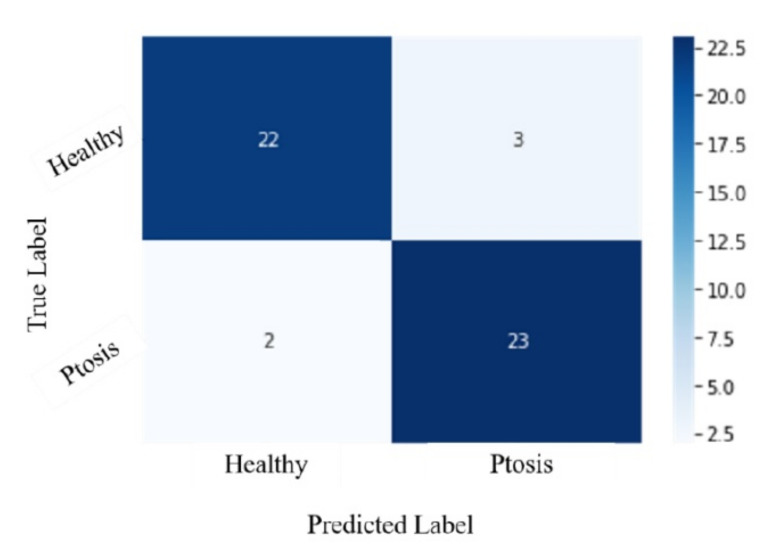
Confusion matrix. The threshold is 0.5.

**Figure 4:**
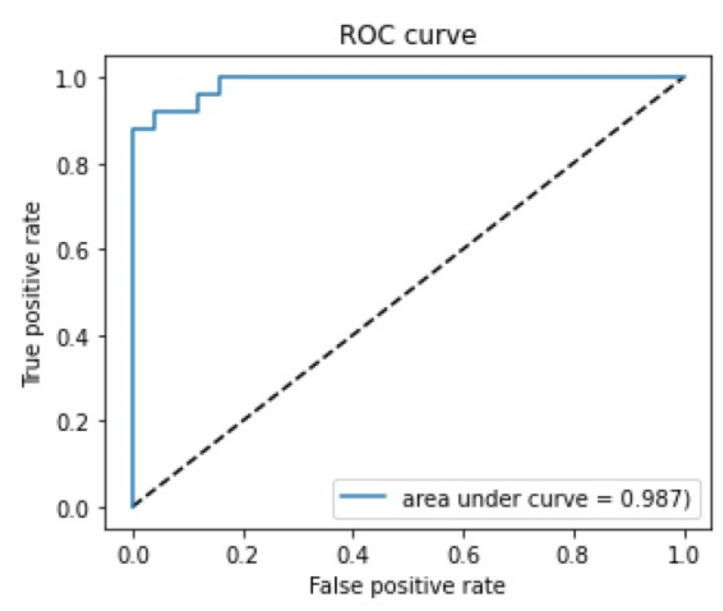
ROC curve. The area under the curve (AUC) is 0.987.

**Figure 5:**
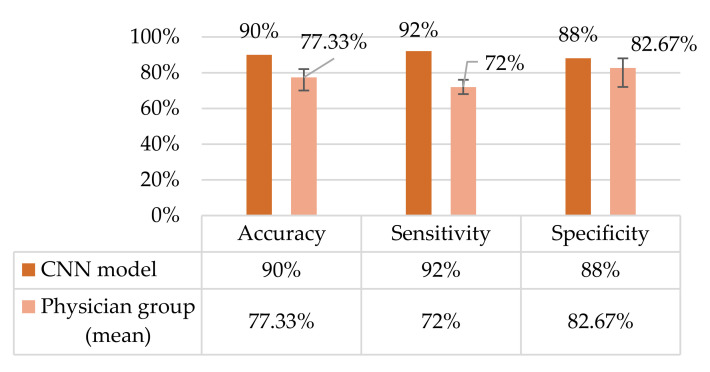
Performance comparison.

The confusion matrix with a 0.5 threshold setting is shown in Figure 3. The receiver operating characteristic (ROC) curve is presented in Figure 4. The area under the curve (AUC) was 0.987. The mean accuracy of the non-ophthalmic physician group was 77.33% (range: 70–82%) with a mean sensitivity of 72% (range: 68–76%) and a mean specificity of 82.67% (range: 72–88%), as seen in Figure 5. 

### 3.2. Grad-CAM Results

**Figure 6:**
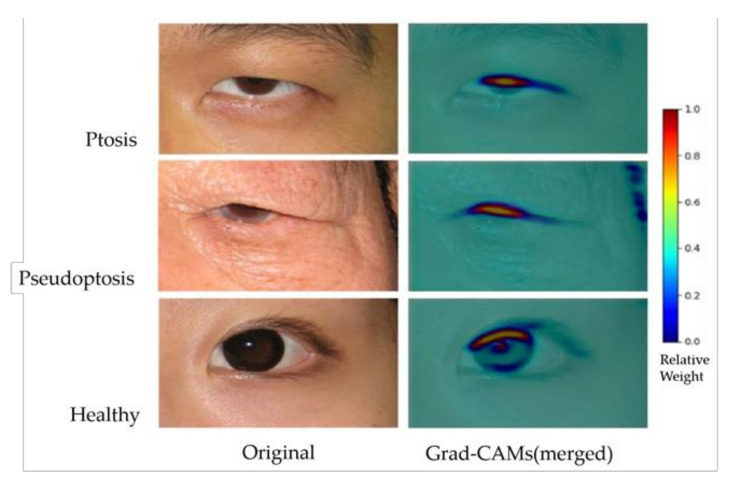
Original images and Grad-CAM results of the AI model predictions. Ptosis (**upper**), pseudoptosis (**middle**), and healthy eyelids (**lower**). Grad-CAM results have been merged with original images.

Gradient-weighted class activation mapping (Grad-CAM) [23] was applied to visualize the AI model. The result showed that the weight in the background was around 0~0.2. In the ptotic eyelids, the area between the upper eyelid margin and the central cornea light reflex showed the highest weight, around 0.5~1.0 (Figure 6).

## 4. Discussion

It is important for general practitioners to promptly diagnose and refer eyelid ptosis, including pseudoptosis, to ophthalmic specialists for further evaluation, work up, and treatment. Pseudoptosis is a heterogeneous group of disorders where the upper eyelid can drop in the absence of pathology of the upper eyelid muscles [24]. Dermatochalasis is likely the most common eyelid condition that causes confusion when evaluating a patient with apparent ptosis. Excess upper eyelid skin may overhang the eyelashes and obstruct the visualization of the eyelid margin, giving the impression of a low-lying eyelid. In a previous proof-of-concept study, we demonstrated that an AI model could detect true ptosis from healthy eyelids [12]. In this study, we evaluated true ptosis and pseudoptosis versus health eyelids, applied a larger dataset of 782 images, and compared the AI model performance to non-ophthalmic physicians. Our results demonstrate that the AI model achieved an accuracy of 90%, with 92% specificity and 88% sensitivity. Additionally, the AI model performed well even when including pseudoptosis cases, which better mimic the real clinical situation in primary care.

A non-ophthalmic group of three physicians, including experts in family medicine, neurology, and emergency medicine, were chosen as a comparator group. The family medicine doctor represented general practitioners who are commonly the first line in seeing and diagnosing age-related and systemic causes of ptosis. The neurologist was selected due to specialized training in diagnosing ptosis, particularly related to neurologic or myogenic causes. Finally, the emergency medicine doctor was selected due to expertise in diagnosing acute causes of ptosis, such as Horner syndrome, third nerve palsy [25], or trauma. Hence, our non-ophthalmic group had previous experience in identifying blepharoptosis. Our results demonstrate a mean accuracy of 77.33% (range: 70–82%), with a mean sensitivity of 72% (range: 68–76%) and a mean specificity of 82.67% (range: 72–88%) in the non-ophthalmic physician group, while the AI model achieved an accuracy of 90%, with a sensitivity of 92% and a specificity of 88%. These results suggest that an AI-aided diagnostic tool can accurately detect blepharoptosis and prompt referral for ophthalmic evaluation when necessary.

CNNs (convolutional neural networks) have achieved great success in image classification. For example, in the current largest image classification dataset classification challenge, ImageNet, all models with top performance used CNN architectures. The general trend is that the deeper the model, the greater discernment the model can provide. Some model structures can be very deep, such as Res Net-152, which has 152 CNN layers. In a previous smaller scale study [12], where less than 500 eyelid pictures were evaluated, a variety of CNN models demonstrated high performance over an accuracy of 80%. This study showed that, for ptosis classification, the most common models, such as VGG, ResNet, AlexNet, SqueezeNet, and DenseNet, all have similar performance. Therefore, among those models, we chose a relatively simple model, VGG-16, a computing resource-efficient model, as our base model. The VGG-16 model is based on architecture developed by the Oxford Visual Geometry Group (VGG) and achieved top performance in the ImageNet Large Scale Visual Recognition Challenge (ILSVRC) 2014.

Gradient-weighted class activation mapping (Grad-CAM) is a visual explanation of the AI model, which is applicable to a wide variety of CNN model-families [23]. To aid the understanding of AI model predictions, a heat map identifies the areas of the input image that contributed most to the AI model’s classification using a technique called class activation mappings. In addition, to visualize reasonable AI predictions, Grad-CAM explanations also helped identify dataset biases in images. For example, a preoperative marking around the eye or a postoperative suture on the eyelid may provide misleading clues to the AI model, rather than eyelid information for blepharoptosis. The results of Grad-CAM (Figure 6) demonstrated a hotspot area (0.5–1.0 in weights) between the upper eyelid margin and central corneal light reflex, which is clinically compatible with the MRD-1 concept. The cold zone (0–0.2 in weights) in the background successfully excluded dataset biases, providing stronger faithfulness. With larger and more diverse data utilization in the future, more precise results to understand the AI predictions can be expected.

AI-assisted ptosis diagnostic tools can be of great impact on the management of congenital ptosis, since up to one-third of congenital ptosis patients are at risk for amblyopia [26]. The accurate diagnosis of ptosis based on external photographs would prove especially helpful in the pediatric population for ophthalmologists and general practitioners alike, as the eyelid exam can be challenging in uncooperative or crying children, patients with developmental delays, and babies. The AI-assisted detection of congenital ptosis could have a huge impact on preventing and treating amblyopia promptly. External validation with outsourced images, including mobile phone photographs, to confirm the strength and weakness of this AI model also deserves further investigation.

Limitations to this study include that the data resource was only from Asian ethnicities, setting limitations in both model training and testing process. Future studies will analyze external photographs from diverse ethnicities to further train the AI model and expand the application for all users. Additionally, only adults were included in this study, setting limitations for pediatric care. Furthermore, we did not measure variables including palpebral aperture, levator muscle excursion, and brow position, which should be identified for detailed and quantifiable ptosis assessment.

There were also inherent limitations in the labeling of the ground truths by three oculoplastic surgeons. Some photos of mild ptosis were challenging to differentiate from normal eyelids, even among experienced oculoplastic surgeons. Hence, the AI model constructed in our study only provided information as to whether a photo might have ptosis from an oculoplastic surgeon’s point of view. This might also explain why this study did not achieve much greater accuracy then our previous study, since more data may introduce more photos with uncertainty [12].

## 5. Conclusions

The AI model using CNNs achieved better performance than the non-ophthalmic physician group and shows value as a diagnostic tool to be used in assisting the referral of blepharoptosis, including true and pseudoptosis.

## Figures and Tables

**Table 1:** The number of images in the training, validation, and testing set.

	Training	Validation (for Training)	Testing
Referable ptosis group	455	113	25
Healthy group	132	32	25

**Table 2:** Structure of the model.

Input Size	Layer	Output Size	Number of Feature Maps	Kernel Size	Stride	Activation
-	Image	200 × 300 × 3	-	-	-	-
200 × 300 × 3	Convolution	200 × 300 × 64	64	3 × 3	1	ReLU
200 × 300 × 64	Convolution	200 × 300 × 64	64	3 × 3	1	ReLU
200 × 300 × 64	Max pooling	100 × 150 × 64	64	-	2	-
100 × 150 × 64	Convolution	100 × 150 × 128	128	3 × 3	1	ReLU
100 × 150 × 128	Convolution	100 × 150 × 128	128	3 × 3	1	ReLU
100 × 150 × 128	Max pooling	50 × 75 × 128	128	-	2	-
50 × 75 × 128	Convolution	50 × 75 × 256	256	3 × 3	1	ReLU
50 × 75 × 256	Convolution	50 × 75 × 256	256	3 × 3	1	ReLU
50 × 75 × 256	Global max pooling	1 × 256	-	-	-	-
1 × 256	Fully connected	1 × 512	-	-	-	ReLU
1 × 512	Fully connected	1	-	-	-	Sigmoid

## Data Availability

The data presented in this study are available on request from the corresponding author. The data are not publicly available due to privacy issue.
